# Network pharmacology combined with pharmacodynamics revealed the anti-inflammatory mechanism of Tanreqing capsule against acute-exacerbation chronic obstructive pulmonary disease

**DOI:** 10.1038/s41598-022-18326-1

**Published:** 2022-08-17

**Authors:** Xiao-Xiao Han, Yan-Ge Tian, Xue-Fang Liu, Di Zhao, Xue-Hang Du, Hao-Ran Dong, Su-Xiang Feng, Jian-Sheng Li

**Affiliations:** 1 College of Pharmacy, Henan University of Chinese Medicine, Zhengzhou, Henan China; 2 Academy of Chinese Medical Sciences, Henan University of Chinese Medicine, Zhengzhou, Henan China; 3 Collaborative Innovation Center for Chinese Medicine and Respiratory Diseases Co-Constructed by Henan Province and Education Ministry of P. R. China, Zhengzhou, 450046 Henan China; 4 Shanghai Kaibao Pharmaceutical Co. Ltd, Shanghai, China; 5 The First Affiliated Hospital, Henan University of Chinese Medicine, Zhengzhou, Henan China

**Keywords:** Diseases, Medical research

## Abstract

Acute-exacerbation chronic obstructive pulmonary disease (AECOPD) is mainly associated with acute respiratory tract infection. In recent years, a growing number of studies have found that Tanreqing capsule (TRQ) has a favorable anti-inflammatory effect. In this study, we used network pharmacology and pharmacodynamics to explore the molecular mechanism and effects of TRQ in AECOPD treatment. To further understand the molecular mechanism of TRQ in AECOPD treatment, we used the network pharmacology to predict components of TRQ, TRQ-related targets, AECOPD-related targets, and pathways. In addition, we used the cigarette-smoke/lipopolysaccharide -induced AECOPD experimental model in Sprague–Dawley rats (72 rats randomly divided into six groups [*n* = 12 each]: control, model, high-TRQ [TRQ-H], medium-TRQ [TRQ-M], low-TRQ, and dexamethasone [Dex]) to evaluate the therapeutic effects of TRQ and to verify the network pharmacology. We found that 59 overlapping targets based on component-and AECOPD-related targets were frequently involved in the advanced glycation end product–receptor for advanced glycation end product signaling pathway in diabetic complications, the phosphatidylinositol-3-kinase–protein kinase B signaling pathway, and the hypoxia-inducible factor 1 signaling pathway, which might play important roles in the anti-inflammatory mechanism of TRQ in AECOPD treatment. Moreover, TRQ groups exerted protective effects against AECOPD by reducing the infiltration of inflammatory cells. Meanwhile, TRQ-M and TRQ-H groups significantly downregulated or upregulated the expression of tumor necrosis factor, interleukin (IL) 6, C-reactive protein, IL10, and serum amyloid A, as key targets in network pharmacology, in the serum and bronchoalveolar lavage fluid to achieve anti-inflammatory efficacy. Our study showed that TRQ had better anti-inflammatory efficacy against AECOPD, and initially elucidated its molecular mechanism. Moreover, our study also provides a new strategy to explore effective mechanism of TRQ against AECOPD; and further studies are needed to validate the biological processes and pathways of TRQ against AECOPD.

## Introduction

Chronic obstructive pulmonary disease (COPD) is characterized by persistent respiratory symptoms and airflow limitation due to airway and/or alveolar abnormalities^1^. On the basis of clinical and pathological characteristics, COPD is divided into stable COPD and acute-exacerbation COPD (AECOPD). AECOPD is manifested as acute worsening of COPD, which has decreased quality of life and increased mortality^2^. AECOPD is caused by various pathogenic factors inside and outside of the lungs, including acute respiratory tract infection, pollutants, cold weather and irritants^3^, and characterized by airway inflammation and mucus hypersecretion^4^. The inflammatory response may induce parenchymal tissue destruction, resulting in emphysema, and disruption of normal repair and defense mechanisms, and small-airway fibrosis^2^. AECOPD is mainly treated by reducing inflammation, such as Tanreqing Injection^5^ and glucocorticoid^6^.

Tanreqing capsule (TRQ) (Z20130025), a widely used classical compound herbal recipe, consists of Scutellariae radix (SR, *Scutellaria baicalensis* Georgi), Bear Bile Powder (BBP, Selenaretos thibetanus Cuvier), Cornu Caprae Hicus (CCH, Naemorhedus goral Hardwicke), Lonicerae japonicae flos (LJF, *Lonicera japonica* Thunb.), and Forsythiae fructus (FF, *Forsythia suspensa* (Thunb.) Vahl), and is accordant with formulation of Tanreqing Injection for treating syndromes including fever, cough and expectoration. The relevant studies showed that Tanreqing Injection has been successful applied into the treatment of AECOPD patients. Liu et al. indicated Tanreqing Injection possesses potent exhibitory effects in LPS-induced airway inflammation by suppressing the MAPKS and NF-κB signaling pathways to downregulate the TNF-α, IL1β, IL6 and IL8 levels in BALF and serum, which attenuate the airway inflammation, airway damage, and mucus hypersecretion^7^. And the previous study indicated that Tanreqing Injection down-regulates the levels of IL-8 and neutrophil elastase (NE) to inhibit the airway inflammation and mucus hypersecretion with satisfactory clinical efficacy in the AECOPD treatment^4^, and reduces the level of inflammatory factors to improve arterial oxygen partial pressure of AECOPD patients^8^. In addition, modern pharmacological studies have revealed that TRQ has anti-inflammation, immunomodulation, antibiosis and antivirals^9^. Fan et al. indicated that TRQ has a good clinical effect on acute bronchitis (cough of wind -heat invading the lung), and shows no toxic and side effects^10^. And, TRQ significantly decreased the transforming growth factor (TGF) β1, IL6 and CRP expression to improve lung function and inhibit inflammatory response against radiation pneumonitis^11^. Therefore, we predicted that TRQ was effective on treating AECOPD, few studies have systematically investigated the molecular mechanism and efficacy of TRQ in AECOPD treatment. In order to confirm this prediction, we investigated the effect and anti-inflammatory molecular mechanism of TRQ on AECOPD rats.

**Figure 1:**
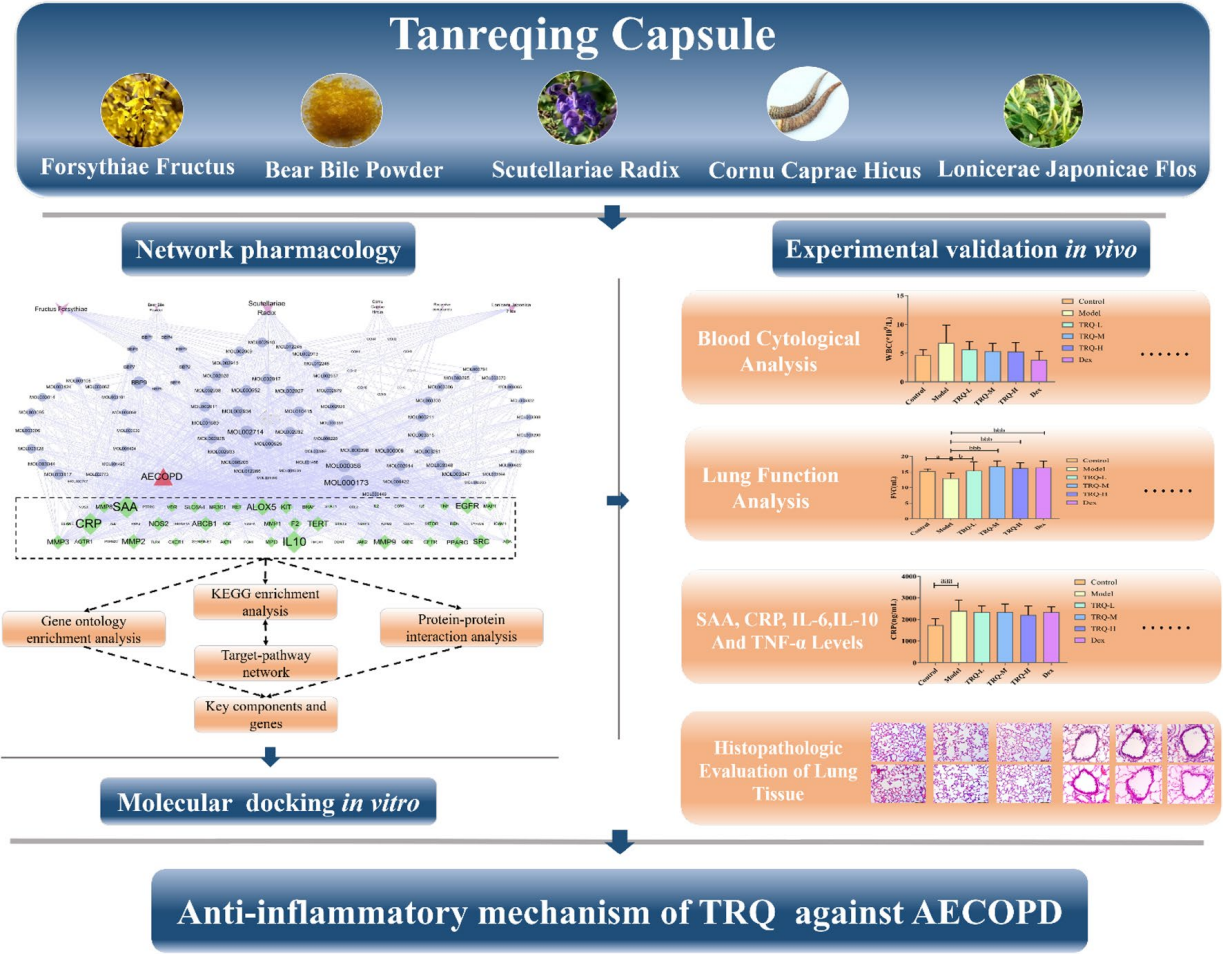
The workflow of the network pharmacology combined with the Pharmacodynamics framework.

Network pharmacology, as a system biology method, is applied to study the mode of action of Chinese medicines (CM). The biological network-based framework for understanding the mechanism of Chinese herbal formular has been established for the first time in 2007, and the first international standard of network pharmacology, Network Pharmacology Evaluation Method Guidance, has been formulated in 2021^12,13^. Network pharmacology can reveal the pharmacological action and molecular mechanism of Chinese medicines and prescriptions through computer algorithms, pharmacological analysis and network database retrieval^12^. Due to the complexity and variety of components involved, research on CM formulas is relatively difficult. Moreover, the holistic characteristic and rich experience of CM highlights the limitations of the reductionist medical research mode, and brings forth a new generation of studies featuring network. Recently, Network pharmacology is developing towards a research model that combines computational, experimental and clinical approaches^14,15^. The development of network pharmacology in recent years has made it possible to investigate CM formulas. In this study, we undertake a systematic study of the efficacy and the possible mechanism of TRQ in AECOPD treatment based on AECOPD animal model and molecular mechanism using network pharmacology. This involves, firstly, we systematically incorporate active compounds prediction, therapeutic targets prediction, and component-target-disease network analysis by network pharmacology. Then, in vitro molecular docking and in vivo pharmacodynamic experiments were used to verify the results of network pharmacology and to evaluate the efficacy of TRQ against AECOPD. The workflow is shown in Fig. 1.

## Materials and methods

### Materials

TRQ was supplied by Shanghai Kaibao Pharmaceutical Company Ltd. (batch: 1911102; specification: 0.4 g/piece; usage and dosage: 3 piece/times and 3 times/day). Dexamethasone pieces were purchased from Zhejiang Xianju Pharmaceutical Company Ltd. (batch:191067; specification: 0.75 mg * 100 pill; usage and dosage: 0.75–3 mg/times and 2–4 times/day; experimental usage and dosage: 0.75 mg/times and 3 times/day). Whole value grain feed stuff was purchased from SPF (Beijing) Biotechnology Company Ltd. (SCXK (Jing) 2019-0010). Lipopolysaccharide (LPS) (batch: L2880) was obtained from Sigma-Aldrich Company Ltd. Klebsiella pneumoniae (KP, 46,114) was obtained from National Center for medical Culture Collections (Beijing, China). An enzyme linked immunosorbent assay (ELISA) kit used to determine the levels of IL6, IL10 and TNF-α was from Wuhan BOSTER Biological Technology Company Ltd. The levels of SAA and CRP were quantified using the ELISA kit from Elabscience Company Ltd. Paraformaldehyde was from Tianjing Chemical Reagent Company Ltd. Ultra-pure water was prepared with a Milli-Q water purification system (Bedford, France).

Bioactive compounds of three herbs in TRQ (SR, LJF, and FF) were collected from the Traditional Chinese Medicine Systems Pharmacology (TCMSP) database (http://tcmspw.com/). Due to the complexity and variety of components, Oral bioavailability (OB) combined with drug likeness (DL) property evaluation were applied to screen the active substance of TCM^16^. OB ≥ 30% and DL ≥ 0.18 were set as screening thresholds to collect the components^17^. The constituents of two animal drugs, including BBP and CCH, were selected from the literature^18–21^. Targets corresponding to the components were derived from the Swiss Target Prediction database (http://www.swisstargetprediction.ch/) combined with the PubChem database (https://pubchem.ncbi.nlm.nih.gov/). The organism was chosen as “Homo sapiens,” and 1357 targets of components were obtained. AECOPD associated targets were gathered from the GeneCard database (https://www.genecards.org/) and the Online Mendelian Inheritance in Man (OMIM) database (https://omim.org/), which were searched using the keywords “acute-exacerbation of chronic obstructive pulmonary disease”.

### Network construction and molecular docking

A total of 2034 associated targets of AECOPD were gathered, and we found 139 AECOPD targets with a median score of ≥ 38.25. To find 59 targets of TRQ treatment for AECOPD from Venny obtained using the Bioinformatics analysis platform (http://www.bioinformatics.com.cn/) (Fig. S1), which were entered into the STRING database for protein–protein interaction (PPI) analysis, a TSV file was downloaded, and Cytoscape 3.2.1 software (Cytoscape Consortium, National Institute of General Medical Sciences, USA) was used to construct TRQ–component–target–AECOPD network and PPI network. Finally, the Metascape database (https://metascape.org/), was used to analyze Gene ontology (GO), including biological processes (BP), cellular components (CC), and molecular functions (MF), and the Kyoto encyclopedia of genes and genomes (KEGG) pathway enrichment^22–24^ of 59 overlapping targets. The targets- pathways map was plotted by http://www.bioinformatics.com.cn, an online platform for data analysis and visualization.

To further predict the interaction of key components and targets of TRQ treatment for AECOPD, the structures of components were collected from the PubChem database and the proteins of targets downloaded from the RCSB PDB (http://www.rcsb.org/), being powerful new tools for exploring 3D macromolecular structures. The above proteins were put into Python software to remove the effect of water and add hydrogen atoms. Finally, molecular docking established by Autodock Tools software was used to validate the network pharmacology results in vitro, moreover, the molecular docking results were visualized by Discovery Studio software.

### Animal experiments

#### Animals and the AECOPD model

Sprague–Dawley rats (36 male and 36 female; body weight 260–300 g) (NO.: 1107261911004350), specific-pathogen-free grade, were purchased from the Animal Experimental Center of Shandong (Shandong, China, SCXK 2019–0003). The experimental protocol was reviewed and approved by the Experimental Animal Care and Ethics Committee of the First Affiliated Hospital, Henan University of Chinese Medicine (Henan, China, SYXK(Yu) 2015–0005). Animal studies were conducted in accordance with the Animal Research: Reporting of in Vivo Experiments (ARRIVE) guidelines^25^, and all breeding and research on experimental animals strictly abide by the regulations on the administration of experimental animals in Henan province. The rats were acclimatized to laboratory conditions for a week before experiments. They were kept in polypropylene cages, maintained under standard conditions of an alternating 12 h light–dark cycle, at a constant temperature of 23 ℃ ± 2 ℃ and a relative humidity of 50%–60%, with free access to water and normal chow. Finally, 72 rats were randomly divided into the following six groups (n = 12 each): control, model, low-TRQ (TRQ-L), medium-TRQ (TRQ-M), high-TRQ (TRQ-H), and dexamethasone (Dex).

Commercially available, filtered Hong Qiqu cigarettes (15 cigarettes/each time, with 10 mg tar, 10 mg nicotine, and 11 carbon monoxide per cigarette (China Tobacco Henan Industrial Company, China)) were used in the early stage of establishing the AECOPD model. The model, TRQ-L, TRQ-M, TRQ-H, and Dex groups were exposed to cigarette smoke with 3000 ± 500 ppm for 3 h each time, twice a day, for 12 weeks^26^. Then, the rats aged 1–8 weeks, received nasal instillation of a *Klebsiella pneumoniae* (KP) suspension (6 × 10^8^ CFU) per week for up to 60 days. Finally, the model, TRQ-L, TRQ-M, TRQ-H, and Dex groups were administrated LPS by intratracheal instillation (2 mg/kg) to induce AECOPD on the first day of the thirteenth week. The KP and LPS doses were selected as previously described^27^. And control group was kept in polypropylene cages with free access to water and normal chow.

The TRQ-L, TRQ-M, and TRQ-H groups were administrated low-, medium-, and high-TRQ doses, respectively, by intragastric administration (0.19, 0.38, and 0.76 g/kg, respectively; formula: Dose (rat) = Dose (human)*6.3*0.5/1/2 = (3.6 g/d)/60 kg*6.3*0.5/1/2 = 0.19/0.38/0.76 g/kg/d) for 7 days (Table 1). The Dex group received an intragastric administration of dexamethasone (2.3 × 10^–4^ g/kg; formula: Dose (rat) = Dose (human)*6.3 *0.5/1/2 = (2.25 mg/d)/60 kg*6.3*1 = 2.3 × 10^–4^ g/kg/d) for 7 days. And control and model groups were intragastrically administrated ultra-pure water (10 mL/kg/d) for the same amount of time. Food was prohibited for 12 h prior to the experiment while water was given freely. Blood was collected from the caudal vein and infrarenal aorta. The serum was separated for further analysis.

**Table 1:** The information of dosage.

Group	Dosage (g/kg/d)	Multiple equivalent dosage with human	Concentrations (mg/mL)	Administration	Volume of administration (mL/kg)	Time of administration (day)
Control	0			Intragastric administration	10	7
Model	0					
TRQ-L	0.19	0.5	19			
TRQ-M	0.38	1.0	38			
TRQ-H	0.76	2.0	76			
Dex	2.3 × 10^–4^	1.0	0.023			

#### Blood cytological analysis

Blood samples taken from the caudal vein were used to detect the white blood cell (WBC) count, the neutrophil cell ratio (NEU%), the lymphocyte ratio (LYM%), and the monocyte ratio (MONO%).

#### Lung function analysis

The rats were anesthetized by intraperitoneal injection of 10% urethane (1.0 mL/100 g), and tracheal intubation was performed to measure the forced vital capacity (FVC), forced expiratory volume at 100 ms (FEV0.1), forced expiratory volume at 300 ms (FEV0.3), peak expiratory flow (PEF), maximum mid-expiratory flow (MMEF), and functional residual capacity (FRC) to assess changes in the lung function of AECOPD rats before and after TRQ treatment.

#### CRP, SAA, IL6, and IL10 levels

On the basis of previous studies and combined with PPI analysis, C-reactive protein (CRP), serum amyloid A (SAA), interleukin (IL)6, IL10, and TNF-α selected as biomarkers were quantified to evaluate the therapeutic effects of TRQ in AECOPD treatment. Blood samples collected from the infrarenal aortic were stewed for 2 h and centrifuged at 12,000 rpm for 15 min, and the supernatant was collected to determine the expression levels of CRP, SAA, IL6, and IL10 using the enzyme-linked immunosorbent assay (ELISA) kit according to the manufacturer’s instructions.

#### TNF-α levels in BALF

The left lung was pumped back and forth gently three times to collect the bronchoalveolar lavage fluid (BALF). The BALF was centrifuged at 12,000 rpm for 15 min, and the TNF-α levels in the supernatant were quantified using the ELISA kit according to the manufacturer’s instructions.

#### Histopathologic evaluation of lung tissue

The left lung tissue was harvested. Samples were fixed in 4% paraformaldehyde, dehydrated, paraffin-embedded, into 4 μm sections, and then stained with hematoxylin and eosin (H&E). The wall thickness (Wt), mean linear intercept (MLI), and mean alveolar number (MAN) were calculated to determine the pathological changes by light microscopy. The schematic diagram of measuring bronchoalveolar wall thickness was that three long-diameters including c1, c2 and c3 and short-diameters including d1, d2 and d3 across the middle of bronchoalveolar were measured under 400blens, the formular of calculating wall thickness(μm) = [(c1−d1) + (c2−d2) + (c3−d3)]/(3 × 2)), as shown in Fig. S2. Finally, the alveolar size and density were calculated by MLI and MAN to assess pathological changes.

### Statistical analysis

Statistical analysis was carried out by one-way analysis of variance using SPSS Statistics 26.0. Least significant difference analysis was applied to groups that conformed to the homogeneity test of variance, while Dunnett’s T3 test was performed for groups inconsistent with the homogeneity test of variance. All data were expressed as the mean ± standard deviation, and α < 0.05 was considered statistically significant.

## Results

### Network pharmacology analysis

**Figure 2:**
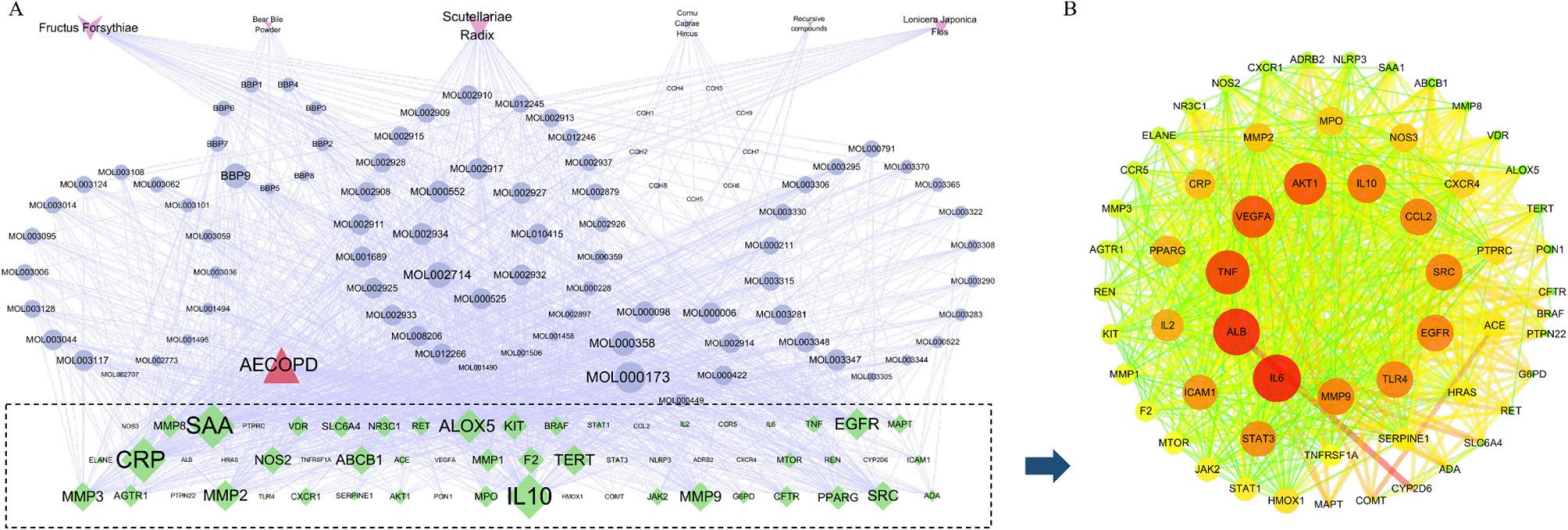
Network pharmacology prediction for TRQ treatment of AECOPD. Analysis of the network of TRQ-component-target-AECOPD including 156 nodes and 1351 edges, the PPI network containing 59 nodes and 682 edges. (**A**) The components of SR (31 in total), LJF (16 in total), FF (18 in total), BBP (9 in total), CCH (9 in total) and CF (7 in total) were marked in blue, disease targets (59 in total) in green, respectively. The size of nodes from large to small represented the degree from large to small. (**B**) The PPI network of the target genes (59 in total) of TRQ treatment for AECOPD. The size of each label represented its degree, the color represented its degree, and thickness and the color of lines represented edge betweenness.

Network pharmacology was used to predict TRQ components and overlapping targets, and to obtain GOBP, GOCC, GOMF, and related signaling pathways, which provided direction to further study the molecular mechanism of TRQ. BBP and CCH are constituents of animal drugs, and the components were not retrieved from the TCMSP database. We found that nine components of BBP had 371 potential targets^20,21^. The components of CCH were similar to those of Saiga tatarica Linnaeus, containing phospholipids, polypeptides, and amino acids^18,19^. Nonetheless, we found that only nine components of CCH had 135 potential targets. We collected 35, 23, and 22 components from SR, FF, and LJF according to screening conditions. The relevant targets of 90 components, in which luteolin, quercetin, kaempferol, wogonin, flavanone, and stigmasterol were shared by 2 or 3 herbs, obtained 1357 results. For example, luteolin was shared by LJF and FF. The component information of herbs is described in Table S1. To understand the complex interaction of components and their corresponding targets, we constructed the TRQ–component–target–AECOPD network (Fig. 2A)^12^. And the network included 156 nodes (5 herbs, 90 components,59 targets, 2 other nodes) and 1351 edges. The mean degree value (the number of target associated with it) of components was 12.46, which indicated that components regulated multiple targets to achieve therapeutic effects. Specially, five compounds, including wogonin, baicalein, 4’-methoxy-7-hydroxyisoflavone, neobaicalein, and β-sitosterol, which acted on 41, 30, 27, 27, and 25 targets, respectively, become the crucial active compounds for the TRQ due to their important positions in this network.

We found 59 overlapping targets to construct a PPI network (Fig. 2B). The average node degree was 24.4, in which 26 targets (degree > 24.4) were selected as core targets. The core targets, including IL6, albumin (ALB), and TNF (being previously known as TNF-α), vascular endothelial growth factor A (VEGFA), AKT1, and IL10, had topological significance and might play an important role in the molecular mechanism of TRQ in AECOPD treatment.

**Figure 3:**
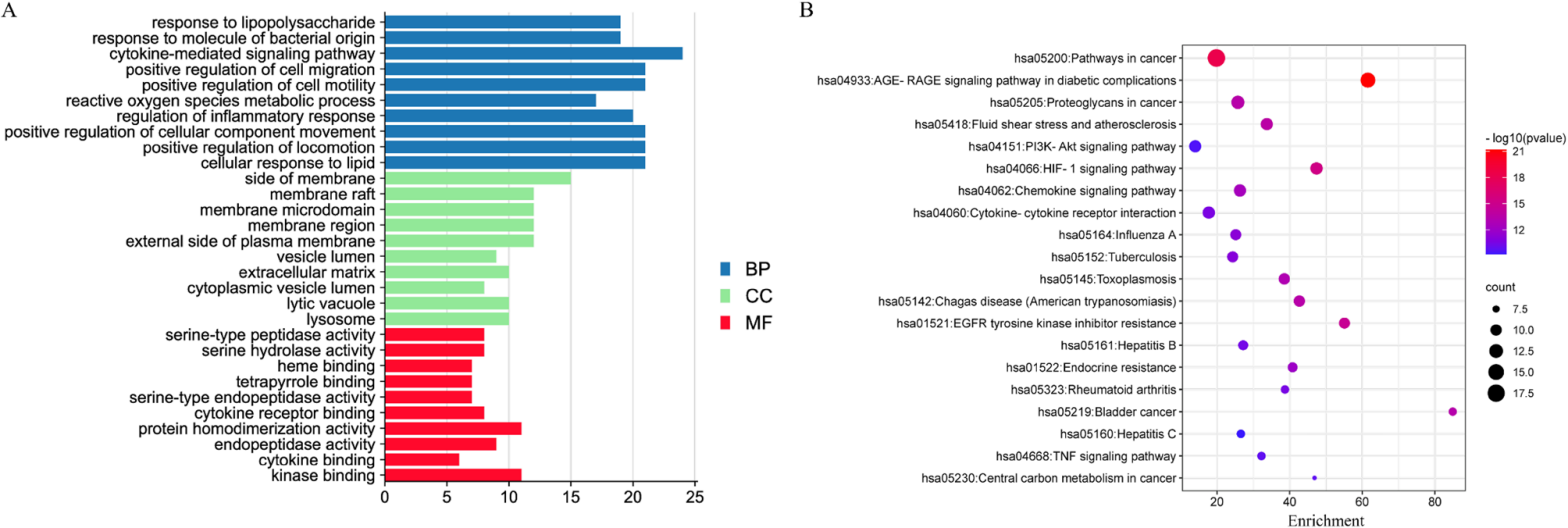
GO and KEGG enrichment analysis of 59 overlapping targets of TRQ for AECOPD treatment. (**A**) GO included GOBP, GOCC and GOMF. (**B)** The size of the bubbles of KEGG represented the gene counts, the color from cold to warm of the bubbles represented the P-value from large to small.

The number of GOBP, GOCC, and GOMF was 1253, 76, and 44, respectively. GOBP, GOCC, and GOMF with all the top 10 of P-values were screened and were represented by a graphical bar with the *P*-value in Fig. 3A. GOBP mainly contained the response to LPS, the response to molecules of bacterial origin, and the cytokine-mediated signaling pathway; GOCC included the side of membrane the membrane raft, and the membrane microdomain; and GOMF consisted of serine-type peptidase activity, serine hydrolase activity, and heme binding.

**Figure 4:**
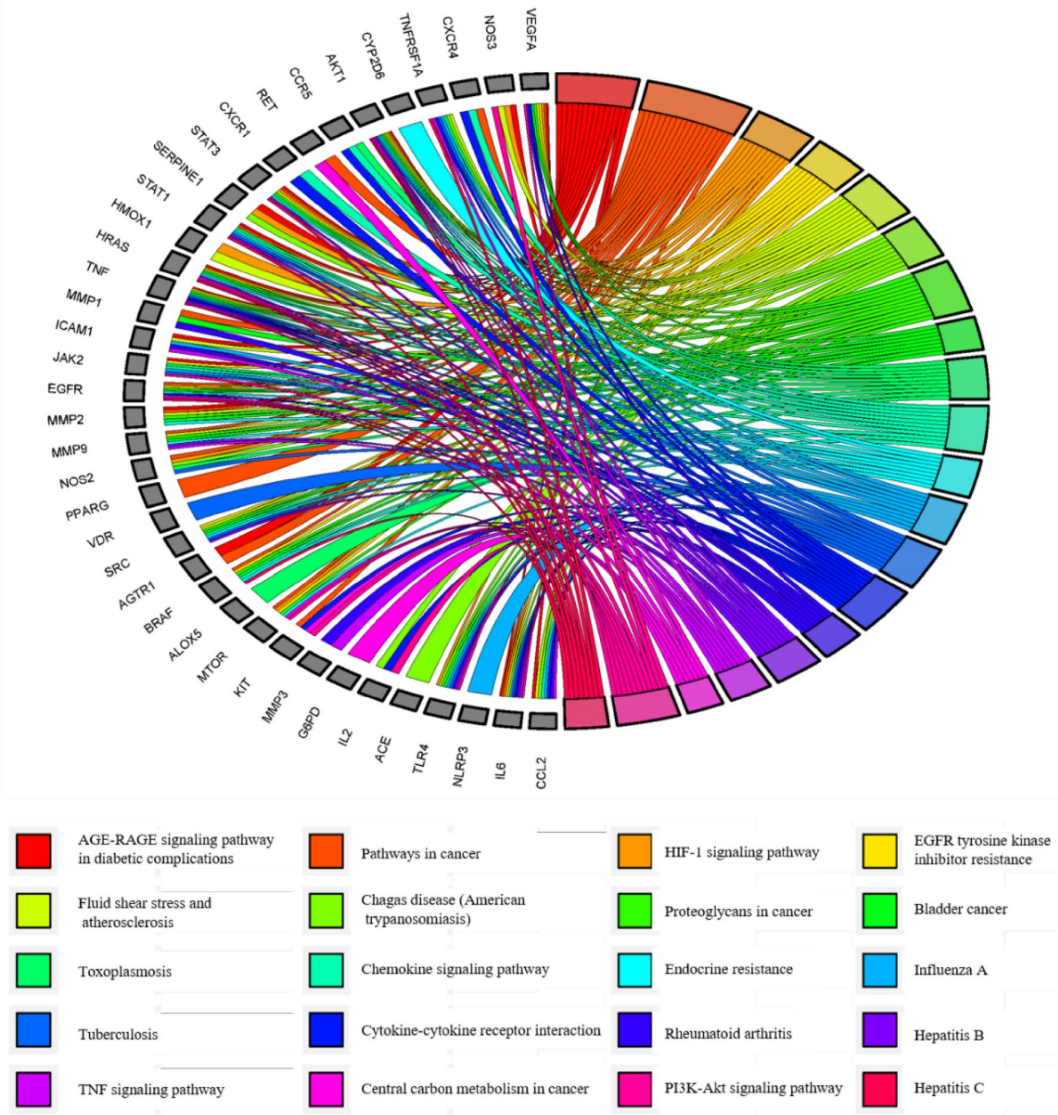
The top 20 pathways of KEGG enrichment analysis for 59 overlapping targets.

Finally, we found 140 related signaling pathways. The top 20 of P-values were represented by a graphical bubble in Fig. 3B, which included the AGE-RAGE signaling pathway in diabetic complications, the PI3K-AKT signaling pathway, and the HIF-1 signaling pathway. Some targets were strongly associated with these signaling pathways, and respectively regulated the above signaling pathways, as shown in Fig. 4.

### Molecular docking

**Figure 5:**
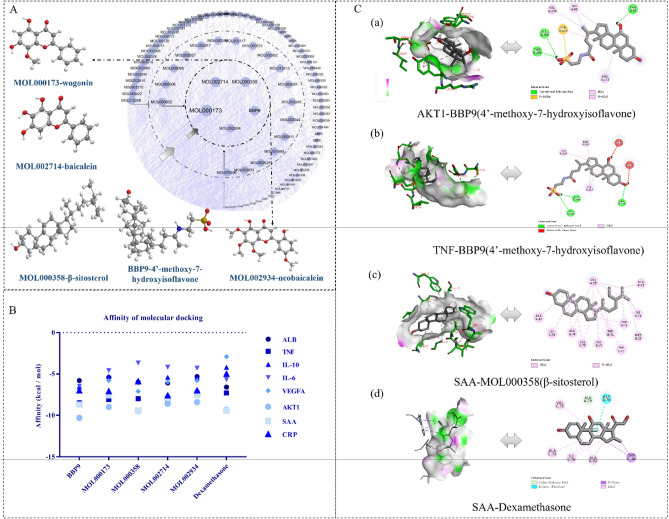
(**A**) The core components, 4’-methoxy-7-hydroxyisoflavone, baicalein, β-sitosterol, wogonin and neobaicalein, were selected from the TRQ-component-target-AECOPD network. (**B**). The affinity results of molecular docking. (**C**). 3D interaction diagrams of key targets and their corresponding best-matched components. (**a**) 3D interaction diagram of 4’-methoxy-7-hydroxyisoflavone in the active sites of AKT1; (**b**) 3D interaction diagram of 4’-methoxy-7-hydroxyisoflavone in the active sites of TNF-α; (**c**) 3D interaction diagram of β-sitosterol in the active sites of SAA; (**d**) 3D interaction diagram of Dexamethasone in the active sites of SAA.

Figure 5A showed that the five core components (wogonin, baicalein, 4’-methoxy-7-hydroxyisoflavone, neobaicalein, and β-sitosterol) selected from the herb–component–target–disease network had a higher degree, and Fig. 5B and Table S2 showed the components and dexamethasone, as positive control in animal experiments, had great affinity with the key targets that had a higher degree in the herb–component–target–disease and PPI networks, including IL6 (PDB ID : 5FUC), ALB (PDB ID : 2BXD), TNF-α (PDB ID : 3WIG), VEGFA (PDB ID : 5T89), AKT1 (PDB ID : 6HHI), SAA (PDB ID : 6PXZ), CRP (PDB ID : 7JME), and IL10 (PDB ID : 4DOH). And 4’-methoxy-7-hydroxyisoflavone possessed the lowest affinity toward TNF-α, IL10, IL6, and AKT1; baicalein possessed the best affinity toward ALB and TNF-α; and β-sitosterol possessed the best affinity toward VEGFA and SAA. In addition, dexamethasone processed the best affinity toward SAA and AKT1.

We drew 3D interaction diagrams of key targets and their corresponding best-matched components to visualize the molecular docking results (Fig. 5Ca–c). Compared with TNF-α, IL10, and IL6, 4’-methoxy-7-hydroxyisoflavone showed a high affinity toward AKT1 (Fig. 5Ca), and the interaction diagram at the active site of the target protein revealed the formation of three hydrogen bonds with ILE290, THR291, and THR82 to stabilize the interaction. Further, the 3D interaction diagram of baicalein at the active site of TNF-α revealed that the interaction was more stable through forming two hydrogen bonds with the key residues ALA333 and SER332 than with CRP and ALB. Finally, the affinity of β-sitosterol toward SAA was higher than toward VEGFA, and its interaction diagram revealed that pi-alkyl stacking interaction occurs with the residues ALA72, ALA79, ALA82, ILE76, VAL75, TRP71, and MET25 and without hydrogen bonds, which contributed to stabilizing the ligand at the active site of the target protein. In addition, the affinity of dexamethasone toward SAA was higher than toward SAA, and its interaction diagram revealed interactions forming one carbon hydrogen bond with the residue ALAA79 and other bonds. The molecular docking results explained the good binding affinity of wogonin, baicalein, 4’-methoxy-7-hydroxyisoflavone, neobaicalein, β-sitosterol, and dexamethasone to some key targets and confirmed the network pharmacology results.

### Animal experiment analysis

#### Blood cytological analysis

**Figure 6:**
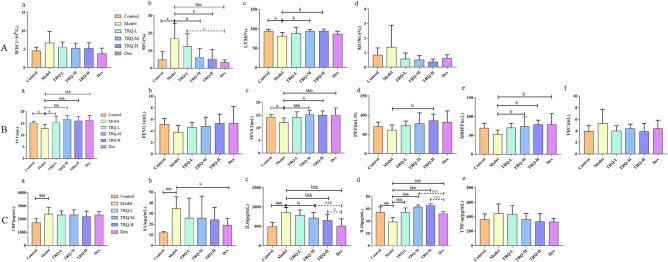
(**A**) Blood cytological analysis in rats to detect the levels of WBC (**a**), NEU (**b**), LYM (**c**) and MONO (**d**). n = 9–12; (**B**). Measuring the relevant indexes of lung function analysis in rats, including FVC (**a**), FEV0.1 (**b**), FEV0.3 (**c**), PEF (**d**), MMEF (**e**), and FRC (**f**). n = 6–12; (**C**). TRQ inhibits inflammatory response in AECOPD rats. TRQ reduced the relevant cytokines level, such as CRP in serum (**a**), SAA in serum (**b**), IL6 in serum (**c**) and TNF-α in BALF (**e**). Moreover, TRQ upregulated the expression level of IL10 in serum (**d**). n = 6–12; ^a^*P* < 0.05, ^aaa^*P* < 0.01 vs. the control group; ^b^*P* < 0.05, ^bbb^*P* < 0.01 vs. the model group; ^c^*P* < 0.05, ^ccc^*P* < 0.05 vs. the Dex group.

Systemic inhalation of cigarette smoke and KP combined with LPS induced AECOPD. Compared to control group, NEU% of rats in the AECOPD model significantly increased (*P* < 0.05), and LYM% dramatically decreased (*P* < 0.05). And compared with the model group, WBCs in the TRQ groups and Dex group decreased with no statistical significance, NEU% significantly decreased in TRQ-M, TRQ-H groups and Dex goup (*P* < 0.05 and *P* < 0.01), LYM% was upregulated in TRQ-M and TRQ-H groups (*P* < 0.05) and in TRQ-L and Dex groups with therapeutic effect, and MONO% decreased in TRQ groups and Dex group without statistical significance, as represented in Fig. 6Aa–d. The results showed that TRQ groups and Dex group could improve AECOPD, in addition, the protective effects of TRQ-M and TRQ-H groups were better than that Dex group for LYM% and MONO%.

#### Lung function analysis

Compared with the model group, FVC increased in the treatment groups (*P* < 0.05 and *P* < 0.01) (Fig. 6Ba); FEV0.3 and MMEF significantly increased in TRQ-M and TRQ-H groups and Dex group (*P* < 0.05 and *P* < 0.01) (Fig. 6Bc,e). TRQ groups and Dex group had protective effects compared with model group in FEV0.1, PEF and FRC without statistical significance, as shown in Fig. 6Bb,d,f. The above results indicated that TRQ groups can improve AECOPD model with therapeutic effect, and TRQ-H had similar protective effects with Dex.

#### TRQ inhibits the inflammatory response in AECOPD

SAA, CRP, IL6, and TNF-α were upregulated, while IL10 was downregulated in the model group compared with the control group, indicating successful modeling of AECOPD. The levels of CRP and TNF-α were downregulated in TRQ groups and Dex group, with no significant differences compared with the model group (Fig. 6Ca,e). Compared with the model group, SAA expression significantly decreased in Dex group (*P* < 0.05) and was downregulated in TRQ groups with therapeutic effect, as shown in Fig. 6Cb. IL10 expression significantly increased in TRQ groups and Dex group compared with the model group (Fig. 6Cd), and the therapeutic effects of TRQ-M and TRQ-H groups were better than that of Dex group (*P* < 0.01). Moreover, compared with the model group, IL6 level was significantly downregulated in TRQ-M and TRQ-H and Dex groups (*P* < 0.05 and *P* < 0.01), as shown in Fig. 6Cc. Therefore, the anti-inflammatory response and protective effect of TRQ-H and TRQ-M are more significant than those of TRQ-L in AECOPD treatment.

#### Histopathologic evaluation of lung tissue

**Figure 7:**
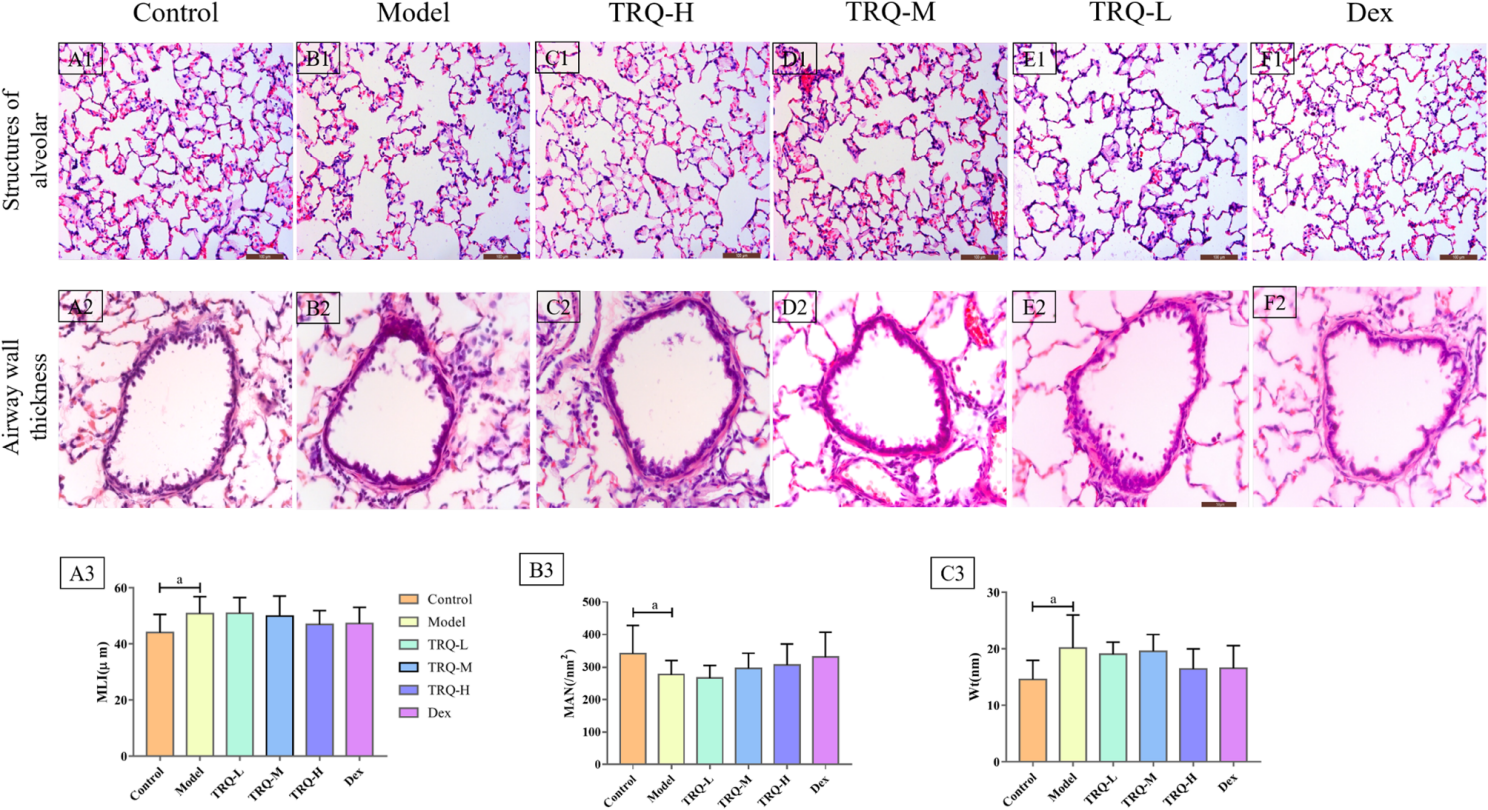
The histopathological changes of lung tissue and effects of TRQ on AECOPD in the rat model. (**A1**–**F1**) Lung tissue sections were stained with hematoxylin and eosin (H&E) for histopathological analysis (magnification 400X), including the structures of alveolar (**A1**–**F1**) and airway wall thickness (**A2**–**F2**). The histopathological changes of lung tissue of control, model, TRQ-H, TRQ-M, TRQ-L and Dex group were represented in **A1** & **A2**, **B1** & **B2**, **C1** & **C2**, **D1** & **D2**, **E1** & **E2** and **F1** & **F2**, respectively. Meanwhile, measuring the relevant indexes included MLI (**A3**), MAN (**B3**) and Wt (**C3**). n = 6–8; ^a^*P* < 0.05 vs. the control group; ^b^*P* < 0.05 vs. the model group.

The model group showed distinct histological changes compared with the control group, including alveolar ectasia, alveolar fusion, airway wall thickening, and infiltration of a mass of inflammatory cells into alveolar spaces and around airway walls (Fig. 7A1,A2,B1,B2). Compared with the model group, pathological changes were significantly alleviated in TRQ-H and Dex groups, including remission of alveolar ectasia and airway wall thickening, normalization of the alveolar wall structure, and few inflammatory cells infiltrating alveolar spaces and around airway walls (Fig. 7C1,C2,F1,F2). Pathological changes were slightly alleviated in the TRQ-M group (Fig. 7D1,D2). In addition, the protective effects of TRQ-L showed no obvious improvement (Figs. 7E1,E2).

Compared with the control group, MLI increased, MAN decreased, and airway wall thickness increased in the model group (*P* < 0.05), as indicated in Fig. 7A3–C3. MLI decreased and MAN increased in TRQ-H, TRQ-M, and Dex groups, with no significant statistical difference, compared with the model group. In addition, airway wall thickness decreased in TRQ-H and Dex groups compared with TRQ-L and TRQ-M. As shown in Fig. 7A3–C3, TRQ groups could improve AECOPD model, and the TRQ-H had similar protective effects with Dex. Therefore, the protective effects of TRQ-H and Dex are better than those of TRQ-M and TRQ-L groups on the structure of alveoli and airway walls.

## Discussion

AECOPD is defined clinically as episodes of increasing respiratory symptoms, particularly dyspnoea, cough and sputum production and increasing sputum purulence^28^. And AECOPD accelerates disease progression and can results in hospital admissions and death. European respiratory society/American Thoracic Society guideline indicated that corticosteroids exert anti-inflammatory to improve lung function in ambulatory patient having a COPD exacerbation^6^. CM characterized as multi-components, multi-channel and multi-target, has the advantages of few side effects, low cost, and low recurring rate, and are widely used to treat AECOPD in the clinic^29,30^. In recent years, CM formulas involved the complexity and variety of components were investigated by network pharmacology combined with molecular docking and in vivo experiments^31^.

Network pharmacology can illustrate the complex interactions among the biological systems, drugs, and disease from a network perspective, which provides new research ideas and technical means to study the mechanisms of action of CM formulae^32^. Due to the complexity of components in TRQ and the diversity of potential targets in humans, we first collected a summary of components and targets of TRQ components and genes associated with AECOPD from multiple databases to further explore the mechanisms underlying the TRQ against AECOPD. Then, the TRQ-component-target-AECOPD network and PPI network were constructed to select the key targets among 59 overlapping targets, including SAA, CRP, IL6, ALB, TNF-α, VEGFA, AKT1, and IL10, which might be the significantly potential targets of TRQ against AECOPD. And TRQ-component-target-AECOPD network also was used to collect the five core components, including 4’-methoxy-7-hydroxyisoflavone, baicalein, wogonin, neobaicalein and β-sitosterol were selected from TRQ-component-target-AECOPD network. The baicalein, neobaicalein and wogonin were from SR, and 4’-methoxy-7-hydroxyisoflavone were from BBP in this study. Moreover, baicalin, wogonin and bile acids are also significant components in Tanreqing Injection by ultra-high-performance liquid chromatography coupled with Q Exactive™ Plus-Orbitrap Fusion mass spectrometry in our previous studies^33^; and 23 bioactive components containing baicalin, wongonin and baincalein etc., in TRQ are also simultaneously quantitated by high-performance liquid chromatography electrospray ionization tandem mass spectrometry^34^. Baicalein, neobaicalein, and wogonin are flavonoids and along with 4’-methoxy-7-hydroxyisoflavone modulate inflammation-associated signaling pathways, thus regulating the expression of pro-inflammatory mediators, and exhibit significant anti-inflammatory activity^35^. Kim et al. investigated the anti-inflammatory effects of 4’-methoxy-7-hydroxyisoflavone after spinal cord injury in rats by inhibiting the expression of the inflammatory cytokines TNF-α, IL1β, and cyclooxygenase-2 (COX-2)^36^. Therefore, the above components might act on 59 overlapping targets to regulate inflammatory-related pathways.

In addition, some targets among 59 overlapping targets are highly enriched inflammatory-related pathways, including AGE-RAGE, PI3K-AKT and HIF-1 signaling pathways. The PI3K-AKT signaling pathway plays a critical role in different cellular processes, including metabolism, inflammation, cell survival, motility, and even cancer^37^. Following activation, PI3Ks, members of the intracellular lipid enzyme family, phosphorylate phosphatidylinositol 4,5- bisphosphate (PIP2), producing phosphatidylinositol 3,4,5-trisphos-phate (PIP3), which recruits AKT into the plasma membrane^38^. The PI3K-AKT signaling pathway is interconnected with several downstream inflammatory, oxidative stress, and apoptotic mediators; therefore, identifying novel multitarget components to attenuate PI3K/AKT regulates related downstream inflammatory cytokines^39^. The links between hypoxia signaling and inflammation are bidirectional, and hypoxia may exacerbate inflammation through activation of inflammatory pathways and affect immune cell fate and function. The HIF-1 signaling pathway intercepts with inflammation, Notch signaling, and stem cell auto renewal and can be activated in normoxia in response to inflammatory stimuli^40^. The results investigated that TRQ regulates inflammatory-related pathways to improve and treat AECOPD.

Molecular docking is an indispensable means to screen active components and calculate the binding affinity of a component to a target, which determines the binding stability. Generally, the lower the affinity, the more stable the binding. Results of molecular docking indicated that AKT1, SAA, TNF, CRP, IL6, IL10 and VEGAF, had significant affinity with the above core components, especially 4’-methoxy-7-hydroxyisoflavone in BBP in our study. Therefore, the above core components could be the pharmacodynamics substances in TRQ that affect the expression of the key targets to treat AECOPD. We initially explored the anti-inflammatory mechanism of TRQ against AECOPD based on network pharmacology, the AECOPD experimental model was used to evaluate the therapeutic potential of TRQ and to verify the network pharmacology in the pharmacological experiment section.

AECOPD is mainly associated with aggravated airway inflammation in previous studies, such as increased levels of acute inflammatory, including SAA, CRP, IL6, IL10, and TNF-α^41^. Quantifying the above cytokines is the current diagnosis and prognosis method for AECOPD. Therefore, the local expression of SAA, CRP, IL6, TNF-α, and IL10 is critical for infiltration of inflammatory cells during AECOPD progression. CRP and SAA levels are the most common indicators used to assess systemic inflammation and curative effects^3^, which shows similar variation tendencies that SAA expression rising 1000 times in 24 h is the same as CRP^39^. Some studies have found that the relative SAA expression is significantly higher in the serum of AECOPD patients compared with stable COPD patients and is positively correlated with and regulated by IL6^42^. SAA and IL6 significantly increase during AECOPD, which effectively predicts the AECOPD process^43^. In addition, some clinical studies also have shown that TNF-α affecting the complex disease process, is higher in AECOPD compared with stable COPD^44^; and IL10 resolves cigarette-smoke-induced inflammatory responses^45^. Moreover, Previous studies indicated that the inhibition of SAA, CRP, IL6 and TNF-α could attenuate AECOPD^41^.

In our research, the AECOPD model in rat, SAA, CRP, IL6 in serum and TNF-α in BALF were significantly upregulated compared with control group. TRQ treatment down-regulated expression of SAA, CRP and IL6 in serum, and TNF-α in BALF. And TRQ treatment, especially TRQ-M and TRQ-H, down-regulated expression of SAA, CRP, IL6, TNF-α. Moreover, the value of IL10, as an anti-inflammatory cytokine, in TRQ-H and TRQ-M was significantly higher than model group, which might imply that TRQ-H and TRQ-M had greater anti-inflammatory capability. In addition, blood cells status in AECOPD model, including the numbers of WBC, NEU% and MONO%, were markedly elevated. The above-mentioned indexes were also improved in the treated groups at different levels. Therefore, our study proved that TRQ improved lung function and decreased systemic inflammation and provided evidence of anti-inflammatory role of TRQ at multiple levels. In our histopathological study, marked inflammatory cell infiltration, alveolar ectasia and fusion, and bronchiolar stenosis were observed in AECOPD rats and were improved in the treated groups at different levels. A high dose of TRQ and dexamethasone has significantly protective effects on the structure of alveoli and airway walls. In addition, the protective effects of high and medium doses of TRQ in alleviating infiltration of inflammatory cells in alveolar spaces and regulating the expression of inflammatory cytokines in AECOPD treatment are better than those of a low dose of TRQ.

Taken together, we demonstrated and proved that TRQ exhibits protective effects on alleviating infiltration of inflammatory cells in alveolar spaces and regulating the expression of SAA, CRP, IL6, IL10, and TNF-α in AECOPD treatment. These findings provided the powerful experimental evidences for the network pharmacology predictions. However, our study initially elucidated its molecular mechanism based on network pharmacology integrating molecular docking and pharmacodynamics, and further studies are needed to validate the biological processes and pathways of TRQ against AECOPD.

## Conclusion

In this study, on the basis of network pharmacology, we systematically revealed molecular mechanism that TRQ can effectively improve the AECOPD-induced inflammatory response. Network pharmacology result preliminarily indicate that TRQ can be used for treating AECOPD by regulating the core targets, including SAA, CRP, IL6, IL10 and TNF-α and relevant signaling pathways (HIF-1, AGE-RAGE, and PI3K-AKT), which lays a foundation for the specific molecular mechanism of TRQ against AECOPD. After analysis and verification by experiment, we experimentally validated that TRQ was effective for the treatment of AECOPD by alleviating infiltration of inflammatory cells in alveolar spaces and regulating the expression of inflammatory cytokines in AECOPD treatment.

## Supplementary Information

Supplementary Information.

## Data Availability

The data was used to support the findings of this study is available. And the results of TRQ fingerprinting based on HPLC-CAD method was attached in Supplementary.

## Abbreviations

TRQ: Tanreqing capsule
AECOPD: Acute-exacerbation chronic obstructive pulmonary disease
LPS: Lipopolysaccharide
AGE-RAGE: Advanced glycation end product–receptor for advanced glycation end product
PI3K-AKT: Phosphatidylinositol-3-kinase/protein kinase B
HIF-1: Hypoxia-inducible factor 1
AKT1: AKT serine/threonine kinase 1
TNF-α: Tumor necrosis factor
CRP: C-reactive protein
SAA: Serum amyloid A
IL: Interleukin
TGF: Transforming growth factor
BALF: Bronchoalveolar lavage fluid
CM: Chinese medicine
NE: Neutrophil elastase
PPI: Protein–protein interaction
TCMSP: Traditional Chinese Medicine Systems Pharmacology
OB: Oral bioavailability
DL: Drug likeness
PPI: Protein–protein interaction
GO: Gene ontology
KEGG: Kyoto encyclopedia of genes and genomes
ELISA: Enzyme-linked immunosorbent assay
VEGFA: Vascular endothelial growth factor A
ALB: Albumin
NF-κB: Nuclear factor kappa B
TCM: Traditional Chinese medicine
SR: Scutellariae radix
BBP: Bear Bile Powder
CCH: Cornu Caprae Hicus
LJF: Lonicerae japonicae flos
FF: Forsythiae fructus
WBC: White blood cell (WBC) count
NEU%: Neutrophil cell ratio
LYM%: Lymphocyte ratio
MONO%: Monocyte ratio
FVC: Forced vital capacity
FEV0.1: Forced expiratory volume at 100 ms
FEV0.3: Forced expiratory volume at 300 ms
PEF: Peak expiratory flow
MMEF: Maximum mid-expiratory flow
FRC: Functional residual capacity
H&E: Hematoxylin and eosin
Dex: Dexamethasone
