# Supplementary material for: Network pharmacology combined with pharmacodynamics revealed the anti-inflammatory mechanism of Tanreqing capsule against acute-exacerbation chronic obstructive pulmonary disease
Source: Sci Rep. 2022 Aug 17;12:13967. doi: 10.1038/s41598-022-18326-1 (PMC9385617; doi:10.1038/s41598-022-18326-1)
Supplement: Supplementary file 1 — Supplementary Information. [file 41598_2022_18326_MOESM1_ESM.docx]

**Table S1.** Information about SR, LJF, FF, BBP and CCH

| **Herb ID** | **MOLID** | **Component** | **OB%** | **DL** |
| --- | --- | --- | --- | --- |
| LJF& FF | MOL000006 | luteolin | 36.16 | 0.25 |
| LJF&FF | MOL000098 | quercetin | 46.43 | 0.28 |
| LJF&FF | MOL000422 | kaempferol | 41.88 | 0.24 |
| SR&FF | MOL000173 | wogonin | 30.68 | 0.23 |
| SR&LJF | MOL002914 | flavanone | 41.35 | 0.24 |
| SR&LJF | MOL000449 | stigmasterol | 43.83 | 0.76 |
| SR&LJF&FF | MOL000358 | β-sitosterol | 36.91 | 0.75 |
| SR | MOL001689 | acacetin | 34.97 | 0.24 |
|  | MOL000228 | alpinetin | 55.23 | 0.2 |
|  | MOL002714 | baicalein | 33.52 | 0.21 |
|  | MOL002908 | 5,8-dihydroxy-2-(2-hydroxyphenyl)-7-methoxychromen-4-one | 37.01 | 0.27 |
|  | MOL002909 | 5,7,2,5-tetrahydroxy-8,6-dimethoxyflavone | 33.82 | 0.45 |
|  | MOL002910 | carthamidin | 41.15 | 0.24 |
|  | MOL002911 | 2,6,2’,4’-tetrahydroxy-6’-methoxychaleone | 69.04 | 0.22 |
|  | MOL002913 | dihydrobaicalin | 40.04 | 0.21 |
|  | MOL002915 | salvigenin | 49.07 | 0.33 |
|  | MOL002917 | viscidulin II | 45.05 | 0.33 |
|  | MOL002925 | 2-(2,6-dihydroxyphenyl)-5,7-dihydroxychromen-4-one | 37.01 | 0.24 |
|  | MOL002926 | (2S)-5,7-dihydroxy-6-methoxy-2-phenyl-2,3-dihydrochromen-4-one | 38.72 | 0.23 |
|  | MOL002927 | skullcapflavone II | 69.51 | 0.44 |
|  | MOL002928 | oroxylin A | 41.37 | 0.23 |
|  | MOL002932 | panicolin | 76.26 | 0.29 |
|  | MOL002933 | 4’-hydroxywogonin | 36.56 | 0.27 |
|  | MOL002934 | neobaicalein | 104.34 | 0.44 |
|  | MOL002937 | dihydrooroxylin A | 66.06 | 0.23 |
|  | MOL000359 | sitosterol | 36.91 | 0.75 |
|  | MOL000525 | norwogonin | 39.4 | 0.21 |
|  | MOL000552 | 5-hydroxy-2-(2-hydroxyphenyl)-6,7,8-trimethoxychromen-4-one | 31.71 | 0.35 |
|  | MOL001458 | coptisine | 30.67 | 0.86 |
|  | MOL001490 | bis(2-ethylhexyl) phthalate | 43.59 | 0.35 |
|  | MOL001506 | squalene | 33.55 | 0.42 |
|  | MOL002879 | diisooctyl phthalate | 43.59 | 0.39 |
|  | MOL002897 | epiberberine | 43.09 | 0.78 |
|  | MOL008206 | moslosooflavone | 44.09 | 0.25 |
|  | MOL010415 | 11,13-eicosadienoic acid, methyl ester | 39.28 | 0.23 |
|  | MOL012245 | 5,7,4’-trihydroxy-6-methoxyflavanone | 36.63 | 0.27 |
|  | MOL012246 | 4’-hydroxywogonin | 74.24 | 0.26 |
|  | MOL012266 | skullcapflavone | 37.94 | 0.37 |
| LJF | MOL003108 | caeruloside C | 55.64 | 0.73 |
|  | MOL003124 | xylostosidine | 43.17 | 0.64 |
|  | MOL003014 | secologanicdibutylacetal | 53.65 | 0.29 |
|  | MOL003101 | 7-epi-vogeloside | 46.13 | 0.58 |
|  | MOL003117 | ioniceracetalides B | 61.19 | 0.19 |
|  | MOL003128 | dimethylmatairesinol | 48.46 | 0.48 |
|  | MOL003006 | (-)-(3R,8S,9R,9aS,10aS)-9-ethenyl-8-(β-D-glucopyranosyloxy)-2,3,9,9a,10,10a-hexahydro-5-oxo-5H,8H-pyrano[4,3-d]oxazolo[3,2-a]pyridine-3-carboxylic acid | 87.47 | 0.23 |
|  | MOL001495 | ethyl linolenate | 46.1 | 0.2 |
|  | MOL003044 | chryseriol | 35.85 | 0.27 |
|  | MOL003095 | corymbosin | 51.96 | 0.41 |
|  | MOL002707 | phytofluene | 43.18 | 0.5 |
|  | MOL002773 | β-carotene | 37.18 | 0.58 |
|  | MOL003059 | kryptoxanthin | 47.25 | 0.57 |
|  | MOL003062 | rhodoxanthin | 31.22 | 0.55 |
|  | MOL001494 | ethyl linoleate | 42 | 0.19 |
|  | MOL003036 | Stigmasterol glucoside | 43.83 | 0.76 |
| FF | MOL000791 | (+)-bicuculline | 69.67 | 0.88 |
|  | MOL003290 | dihydro-β-ionone | 52.3 | 0.48 |
|  | MOL003347 | hyperforin | 44.03 | 0.6 |
|  | MOL003348 | 2-hydroxy-6-methyl-5-(2-methylbutanoyl)-1,3,7-tris(3-methylbut-2-enyl)-6-(4-methylpent-3-enyl) bicyclo [3.3.1] non-2-ene-4,9-dione | 44.03 | 0.61 |
|  | MOL000522 | arctiin | 34.45 | 0.84 |
|  | MOL003283 | isolariciresinol | 66.51 | 0.39 |
|  | MOL003322 | forsythinol | 81.25 | 0.57 |
|  | MOL003365 | lactucasterol | 40.99 | 0.85 |
|  | MOL003370 | onjixanthone I | 79.16 | 0.3 |
|  | MOL003295 | (+)-pinoresinol monomethyl ether | 53.08 | 0.57 |
|  | MOL003306 | forsythin | 85.12 | 0.57 |
|  | MOL003330 | phillygenin | 95.04 | 0.57 |
|  | MOL000211 | Betulinic acid | 55.38 | 0.78 |
|  | MOL003281 | 20(S)-dammar-24-ene-3β,20-diol-3-acetate | 40.23 | 0.82 |
|  | MOL003308 | (+)-pinoresinol monomethyl ether-4-D-β-glucoside | 61.2 | 0.57 |
|  | MOL003315 | 3β-acetyl-20,25-epoxydammarane-24α-ol | 33.07 | 0.79 |
|  | MOL003344 | β-amyrin acetate | 42.06 | 0.74 |
|  | MOL003305 | phillyrin | 36.4 | 0.86 |
| BBP | BBP1 | tauroursodeoxychoilc acid | - | - |
|  | BBP 2 | taurochenodeoxycholic acid | - | - |
|  | BBP 3 | ursodiol | - | - |
|  | BBP 4 | chenodexycholic acid | - | - |
|  | BBP 5 | cholesterol | - | - |
|  | BBP 6 | 4’,7-dihydroxyisoflavone | - | - |
|  | BBP 7 | 4’,7-dihydroxy-6-methoxyisoflavone | - | - |
|  | BBP 8 | 4’,5,7-trihydroxyisoflavone | - | - |
|  | BBP 9 | 4’-methoxy-7-hydroxyisoflavone | - | - |
| CCH | CCH1 | proline | - | - |
|  | CCH2 | citrulline | - | - |
|  | CCH3 | 1-methyhistidine | - | - |
|  | CCH4 | phenylalanine | - | - |
|  | CCH5 | methionine | - | - |
|  | CCH6 | tyrosine | - | - |
|  | CCH7 | histidine | - | - |
|  | CCH8 | tryptophan | - | - |
|  | CCH9 | argininic acid | - | - |

**Table S2.** The affinity results of molecular docking.

| **Affinity(kcal/mol)** | **ALB** | **TNF-α** | **IL-10** | **IL-6** | **VEGFA** | **AKT1** | **SAA** | **CRP** |
| --- | --- | --- | --- | --- | --- | --- | --- | --- |
| BBP9 | -5.8 | -8.5 | -6.5 | -6.5 | -6.9 | -10.3 | -8.7 | -7.0 |
| MOL000173 | -5.4 | -8.1 | -5.3 | -4.6 | -5.8 | -9.0 | -7.6 | -7.1 |
| MOL000358 | -6.0 | -8.0 | -5.8 | -3.7 | -7.1 | -9.5 | -9.4 | -5.9 |
| MOL002714 | -6.1 | -8.5 | -5.4 | -4.2 | -6.0 | -8.6 | -7.9 | -7.6 |
| MOL002934 | -5.3 | -7.3 | -5.4 | -4.3 | -5.8 | -8.4 | -7.5 | -7.0 |
| Dexamethasone | -6.6 | -7.3 | -4.2 | -5.7 | -2.9 | -9.3 | -9.4 | -5.0 |


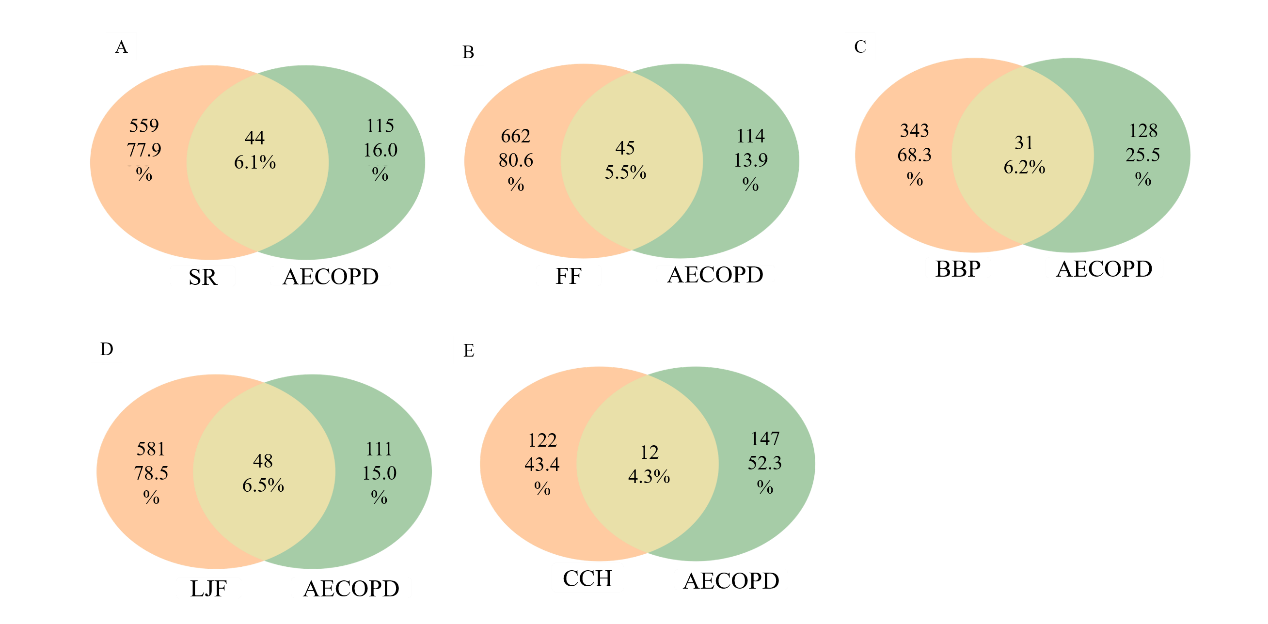


**Figure S1.** The Venny of 59 targets of TRQ treatment for AECOPD. A, B, C, D and E were represented the relevant targets of SR, FF, BBP, LJF and CCH treatment for AECOPD.


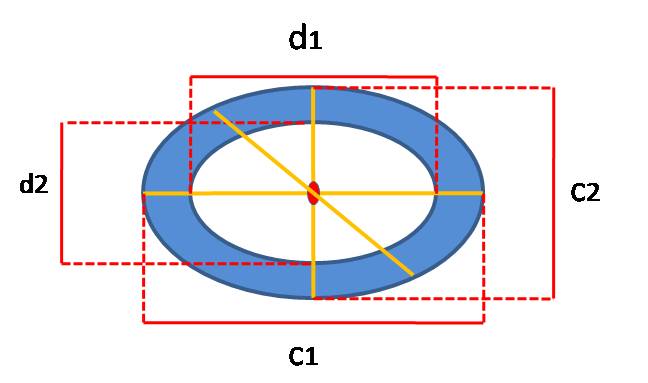


**Figure S2.** The schematic diagram of measuring bronchoalveolar wall thickness.

(Three long-diameters including c1, c2 and c3 and short-diameters including d1, d2 and d3 across the middle of bronchoalveolar were measured under400blens, the formular of calculating wall thickness(μm) = [(c1-d1) + (c2-d2) + (c3-d3)] / (3×2))


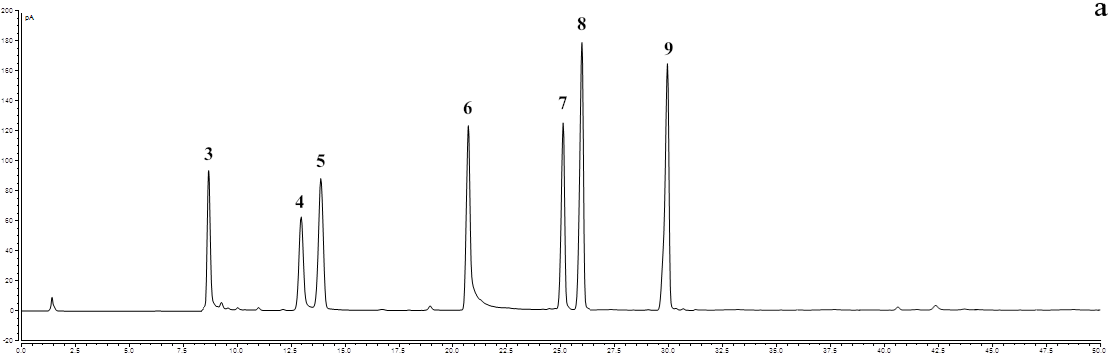


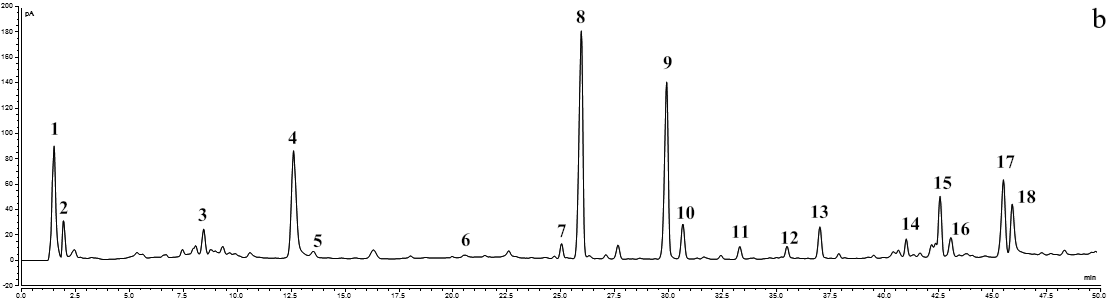


**Figure S3.** The fingerprinting chromatograms of TRQ (a: standard chromatogram; b: sample chromatogram). Peak 3: Forsythoside A, Peak 4: Baicalein, Peak 5: Forsythin, Peak 6: Baicalin, Peak 7: Hyocholic Acid, Peak 8: Ursodeoxycholic acid, Peak 9: Chenodeoxycholic acid.

Method for fingerprinting analysis of TRQ is as follow: Baicalein (batch: CHB190115), Ursodeoxycholic acid (batch: CHB201123), Chenodeoxycholic acid (batch: CHB201217) (purity > 98%) were obtained from Chengdu Chroma-Biotechnology Co., Ltd (Chengdu, China). Forsythoside A (batch: MUST-14060118) and Hyocholic Acid (batch: MUST-14070605) (purity > 98%) were purchased from Chengdu MUST Bio-technology Co., Ltd. Forsythin (batch: Z27A9X59731) and Baicalin (batch: C07M10Y87479) (purity > 98%) were purchased from Shanghai Yuanye Bio-technology Co., Ltd. Each sample was analyzed on HPLC- charged aerosol detector (CAD) (Thermo Scientific, USA) equipped with a UPLC column (Hypersil GOLD 2.1 mm×100 mm, 3 μm). The mobile phase system composed of acetonitrile (A) and 0.1% formic acid in water (B). The flow rat was controlled at 0.2 mL/min with a gradient program of 0-4 min, 8-22%A; 4-10 min, 22-23%A; 10-15 min, 23-33%A; 15-30 min,33-60%A; 30-45 min,60-90%A; 45-50 min,90-95%A. The temperature of column was maintained at 30 ℃ and injection volume was 5μL. And The CAD of optimal conditions was set as follows: evaporation temperature nominal, 35 ℃; collection Rate, 10 Hz; filter constant, 5.0 s. HPLC-CAD method was established to validated 7 components, including Forsythoside A, Baicalein, Forsythin, Baicalin, Hyocholic Acid, Ursodeoxycholic acid, and Chenodeoxycholic acid.
